# Lhermitte’s Sign following VMAT-Based Head and Neck Radiation-Insights into Mechanism

**DOI:** 10.1371/journal.pone.0139448

**Published:** 2015-10-08

**Authors:** Huaising C. Ko, Allison R. Powers, Ren-dih Sheu, Sarah L. Kerns, Barry S. Rosenstein, Stephen C. Krieger, Waleed F. Mourad, Kenneth S. Hu, Vishal Gupta, Richard L. Bakst

**Affiliations:** 1 Department of Radiation Oncology, Icahn School of Medicine at Mount Sinai Hospital, New York, New York, United States of America; 2 Department of Radiation Oncology, University of Rochester Medical Center, Rochester, New York, United States of America; 3 Department of Neurology, Icahn School of Medicine at Mount Sinai Hospital, New York, New York, United States of America; 4 Department of Radiation Oncology, Mount Sinai at Beth Israel Medical Center, New York, New York, United States of America; 5 Department of Radiation Oncology, Albert Einstein School of Medicine at Montefiore Medical Center, New York, New York, United States of America; University of Algarve, PORTUGAL

## Abstract

**Purpose/Objectives:**

We observed a number of patients who developed Lhermitte’s sign (LS) following radiation to the head and neck (H/N), since instituting volumetric modulated arc therapy (VMAT). We aimed to investigate the incidence of LS following VMAT-based RT without chemotherapy, and determine the dosimetric parameters that predict its development. We explored whether the role of inhomogeneous dose distribution across the spinal cord, causing a “bath-and-shower” effect, explains this finding.

**Methods and Materials:**

From 1/20/2010–12/9/2013, we identified 33 consecutive patients receiving adjuvant RT using VMAT to the H/N without chemotherapy at our institution. Patients’ treatment plans were analyzed for dosimetric parameters, including dose gradients along the anterior, posterior, right, and left quadrants at each cervical spine level. Institutional Review Board approval was obtained.

**Results:**

5 out of 33 (15.2%) patients developed LS in our patient group, all of whom had RT to the ipsilateral neck only. LS patients had a steeper dose gradient between left and right quadrants across all cervical spine levels (repeated-measures ANOVA, p = 0.030). Within the unilateral treatment group, LS patients received a higher mean dose across all seven cervical spinal levels (repeated-measures ANOVA, p = 0.046). Dose gradients in the anterior-posterior direction and mean doses to the cord were not significant between LS and non-LS patients.

**Conclusions:**

Dose gradients along the axial plane of the spinal cord may contribute to LS development; however, a threshold dose within the high dose region of the cord may still be required. This is the first clinical study to suggest that inhomogeneous dose distributions in the cord may be relevant in humans. Further investigation is warranted to determine treatment-planning parameters associated with development of LS.

## Introduction

Lhermitte’s sign (LS), a sensation of electrical shock with neck flexion or movement, is a transient phenomenon that correlates with demyelination of the white matter in the spinal cord. Given its intensity and unexpected nature, LS is often an anxiety-provoking experience for patients. LS can present in various disease states, but it is most often noted in patients with multiple sclerosis and occasionally arises as a side effect of radiation to the head and neck. While rare, LS has sometimes preceded the development of irreversible radiation myelopathy, causing paralysis [1].

Radiation is thought to impart injury to the myelin-forming oligodendrocytes in the CNS, causing temporary demyelination until the glial cells can recover [2, 3]. In virtually all cases, the symptoms of LS last for a few months and gradually resolve spontaneously as the oligodendrocytes return to manufacturing myelin. Specifically, radiation-induced LS appears as early as 1 month after radiation treatment and resolves spontaneously around 6 months [4–6]. Previous studies have reported the incidence of LS following conventional radiation therapy or IMRT, with and without chemotherapy, to the head and neck region ranges 3–13% [1, 7–9]. Many chemotherapy agents, such as carboplatin, cytarabine, and others are radiosensitizers and can cause peripheral neuropathies; it is conceivable that radiation exposure in the spinal cord makes the blood-brain barrier more vulnerable to the neurotoxic effects of chemotherapy in the spinal cord. Chemotherapy has been shown in rare instances to cause LS by itself [8, 10, 11], and its contributing effects toward LS in the context of radiation are not well understood. There is currently no data to suggest that surgery to the head and neck region increases the incidence.

Radiogenic LS is a poorly understood phenomenon, but there are a number of leading theories about its underlying mechanism. Previous hypotheses regarding the pathophysiology of radiation-induced LS have included that of a maximum dose threshold. The damaging effect of a larger radiation dose on the spinal cord is undeniably a part of the mechanism leading to LS. Previous studies have found that LS patients were more likely to have cervical cord doses higher than 50 Gy, or more than 2 Gy per fraction [7, 9, 12]. A newer hypothesis of radiogenic LS, extrapolated from the results of animal studies, entails the consequences of small areas of high dose placed in a larger area of low dose radiation, an inhomogenous dose distribution referred to as, “bath-and-shower.” A study by Pak *et al*. prospectively investigated the correlation of LS in 15 of seventy-three head and neck cancer patients with chemo-IMRT dose distributions in a bath-and-shower pattern [13]. The “shower” distribution was defined as a small, high dose area, surrounded by a low-dose, sub-threshold “bath” area. This was analyzed by reviewing the dose-volume histograms (DVHs) of the LS and non-LS patients to look for bath doses of 20 Gy or less surrounding high-dose shower treatment areas. The data from this study supported the previous hypothesis of dose-threshold in that LS was significantly correlated with a higher mean dose, V30, V40 and spinal cord volume receiving ≥ 30 Gy, and ≥ 40 Gy. However, the researchers were unable to correlate LS incidence with the appearance of bath-and-shower distribution patterns analyzed in this manner. A major confounder in this study was the use of concurrent chemotherapy. We believe that this hypothesis is worthy of further investigation in a cohort that did not receive any chemotherapy.

Radiation plans with volumetric modulated arc therapy (VMAT), a newer radiation technique, are highly conformal and with more efficient treatment times compared to IMRT [14]. Since instituting predominately VMAT-based plans to treat head and neck cancer, we observed a number of cases of LS that prompted further investigation. To our knowledge, the incidence of LS following radiation without chemotherapy using modern radiation techniques has never been reported. To better understand the mechanisms of RT-induced LS, we examine the association between inhomogeneous dose patterns in the spinal cord and the development of LS in a cohort that did not receive chemotherapy.

## Materials and Methods

### Patients

From 1/20/2010–12/9/2013, we identified all patients at our institution who received adjuvant radiation treatment to the head and neck region using VMAT. Patients with pre-existing neurological conditions were excluded from the analysis. Since we wished to investigate the effect of VMAT on LS, patients who received chemotherapy at any point during their treatment were excluded to eliminate radiosensitization by chemotherapy as a confounder. In total, n = 33 patients were included in the analysis. Patient characteristics analyzed included: surgery type, maximum and mean dose, tumor histology and stage, treatment of primary tumor ± neck, unilateral or bilateral neck volumes, median time to follow up, and symptom onset and duration.

### Ethics

Our Institutional Review Board specifically approved this study. Patients were not consented because this study was a retrospective review. Patient records and information were anonymized and de-identified prior to analysis.

### Spinal Cord Dosimetry

To investigate the dosimetric differences involved in LS development, the patients were divided into LS and non-LS patients, after which they were further divided for subset analysis. Using dosimetric data from Varian Eclipse treatment planning system, the cervical spinal cord was segmented into seven vertebral levels, which was then divided into 4 quadrants (anterior, posterior, right and left) along the centroid of every CT slice. This division into quadrants was made to distinguish the dose received to varying regions of the cervical spinal cord along the axial plane. Dose gradients were calculated by finding the absolute difference in dose between two of four quadrants (left, right, anterior, and posterior) of a given vertebral level.

### Statistical Analysis

Repeated-measures analysis of variance (ANOVA) was used to test for an overall difference in mean dose and mean dose difference (anterior-posterior sides and left-right sides) comparing patients who developed LS to those who did not develop LS. The repeated-measures ANOVA included dose measurements that were made across seven cervical vertebra levels for each patient, and it tests for both between-subjects effects (comparing dose between LS cases and controls) and within-subjects effects (comparing dose across vertebral levels). One-sided and two-sided t-tests, were also performed to compare clinical characteristics between LS and non-LS groups, and to compare dosimetric parameters at individual vertebral levels. Statistical analysis was performed using R version 3.0.2 (R Foundation for Statistical Computing, Vienna, Austria. URL http://www.R-project.org/). Fisher’s exact test and student’s t-test were used to test categorical and continuous variables respectively for association with LS status.

## Results

The average age of the patients included in this study was 62.3 ± 12.7 years. Most patients in both groups were of male gender (81.8%), and with tumors of squamous cell carcinoma histology (84.8%). Nearly two-thirds of the patients were node-positive (63.6%). A vast majority of patients underwent surgery (93.9%) before treatment with radiation therapy more likely reflecting referral patterns in our department rather than a selection bias. A summary of patient demographics and characteristics can be found in Table 1.

**Table 1:** All patients with VMAT radiation treatment to the head and neck, without chemotherapy.

	All Patients (n = 33)
Age (years)*	62.3 ± 12.7
% Male	81.8% (27)
Histology	
Squamous Cell Carcinoma	84.8% (28)
Acinic Cell carcinoma	3.0% (1)
Adenoid cystic carcinoma	3.0% (1)
Carcinoma ex-pleomorphic adenoma	3.0% (1)
Mucoepidermoid carcinoma	3.0% (1)
Adenosquamous cell carcinoma	3.0% (1)
Primary site	
Oropharynx	51.5% (17)
Oral cavity	12.1% (4)
Parotid	12.1% (4)
Neck	12.1% (4)
Supraglottic larynx	3.0% (1)
Oral tongue	3.0% (1)
Hypopharynx	3.0% (1)
Unknown	3.0% (1)
TNM Stage	
% T0	3.0% (1)
% T1	42.4% (14)
% T2	42.4% (14)
% T3	12.1% (4)
% N0	33.3% (11)
% N1	15.5% (5)
% N2	48.5% (16)
% Nx	3.0% (1)

* Values are represented as mean ± standard deviation, unless noted otherwise

At our institution, we have observed an LS incidence of 15.2% (5 of 33)—exclusively in patients that received unilateral radiation therapy with VMAT. Patients with LS were, on average, younger than their non-LS counterparts (54.2 ± 16.8 years v.s. 64.1 ± 11.9 years), consistent with other studies noting that the incidence of LS is much lower after age 60 [9, 13, 15]. All LS patients had either T1 or T2 tumors. Clinical risk factors for peripheral neuropathy (diabetes, hypertension) among the LS patients consisted of 2 patients with history of hypertension only. The average treatment duration of both LS and non-LS patients was similar (1.6 ± 0.2 and 1.6 ± 0.3 months, respectively). The average time to LS onset was 3.6 ± 2.1 months, with average symptom duration of 7.7 ± 2.5 months. The median follow up time for LS patients was 15.7 months (range: 6.0 to 21.5). A summary of the clinical characteristics of LS and non-LS patients can be found in Table 2.

**Table 2:** Comparison of Lhermitte’s Sign (LS) vs non-LS patients with VMAT radiation to the head and neck, without chemotherapy *.

	LS (n = 5)	Non- LS (n = 28)
Age (years)		
Mean ± standard deviation	54.2 ± 16.8	63.7 ± 11.7
Gender		
% Male	80.0% (4)	82.1% (23)
Primary site		
Oropharynx	80.0% (4)	50.0% (14)
Oral cavity	0% (0)	14.3% (4)
Parotid	20.0% (1)	7.1% (2)
Supraglottic larynx	0% (0)	3.6% (1)
Neck	0% (0)	10.7% (3)
Submandibular gland	0% (0)	3.6% (1)
Oral tongue	0% (0)	3.6% (1)
Hypopharynx	0% (0)	3.6% (1)
Unknown	0% (0)	3.6% (1)
TNM Stage		
% T0	0% (0)	3.6% (1)
% T1	60.0% (3)	39.3% (11)
% T2	40.0% (2)	42.9% (12)
% T3	0% (0)	14.3% (4)
% N0	20.0% (1)	35.7% (10)
% N1	0% (0)	17.9% (5)
% N2	60.0% (3)	46.4% (13)
% Nx	20.0% (1)	0% (0)
Surgery ± neck dissection		
Surgical excision with neck dissection	60.0% (3);	78.6% (22)
Surgical excision without neck dissection	40.0% (2)	7.1% (2)
Unilateral neck dissection only	0% (0)	3.6% (1)
No surgery	0% (0)	10.7% (3)
Radiation treatment		
Treatment to primary only	0% (0)	17.9% (5)
Treatment to primary site + unilateral neck	80.0% (4)	32.1% (9)
Treatment to primary site + bilateral neck	0% (0)	35.7% (10)
Treatment to neck only		
Unilateral neck	20.0% (1)	10.7% (3)
Bilateral neck	0% (0)	3.6% (1)
Unilateral treatment of disease	100% (5)	42.9% (12)
Treatment duration (months)	1.6 ± 0.2	1.6 ± 0.3
Median and range follow up time (months)	15.7 (6.0, 21.5)	7.2 (1.1, 30.3)
Time to LS symptom onset (months)	3.6 ± 2.1	N/A
Symptom duration (months)	7.7 ± 2.5	N/A
Volume receiving		
> 20Gy (cc)	12.0 ± 6.3	10.9 ± 5.9
> 25Gy (cc)	9.8 ± 4.6	9.6 ± 6.0
> 30Gy (cc)	6.7 ± 2.8	7.9 ± 5.8
> 35Gy (cc)	3.5 ± 2.4	5.2 ± 5.3
> 40Gy (cc)	0.7 ± 1.1	1.8 ± 2.8
Mean dose to spinal cord (Gy)	26.1 ± 8.4	24.3 ± 9.0
Maximum dose to spinal cord (Gy)	40.5 ± 3.0	38.8 ± 7.0
Dose per fraction (Gy)	2.0 ± 0	2.0 ± 0.0

* figures are represented as mean ± standard deviation, unless noted otherwise

Patients that developed LS described their symptoms as, “shock-like,” “shooting,” “hair-raising,” and “electric” sensations that were felt in the back or trunk (4 of 5 patients), lower extremities (4 of 5 patients), and upper extremities (1 of 5 patients). In most cases, the sensation was brought on by flexing the neck forward, and was felt bilaterally. At median follow-up time of 15.7 months, the LS symptoms of all but one patient had completely resolved.

With regards to dose volumes in the cord, LS patients had similar, if not smaller, volumes compared to their non-LS counterparts. For volumes receiving > 40 Gy, the LS patients had less than non-LS patients (0.7 ± 1.1 cc v.s. 1.8 ± 2.8, respectively). LS patients had a slightly higher maximum cord dose, compared to non-LS patients (40.5 ± 3.0 Gy and 38.8 ± 7.0 Gy, respectively). All patients received a dose of 2 Gy per fraction, with the exception of one non-LS patient, who received 2.2 Gy. All p-values calculated for these clinical and dosimetric characteristics were greater than p = 0.05. A summary of all comparative dosimetric characteristics between LS and non-LS patients can also be found in Table 2.

**Fig 1:**
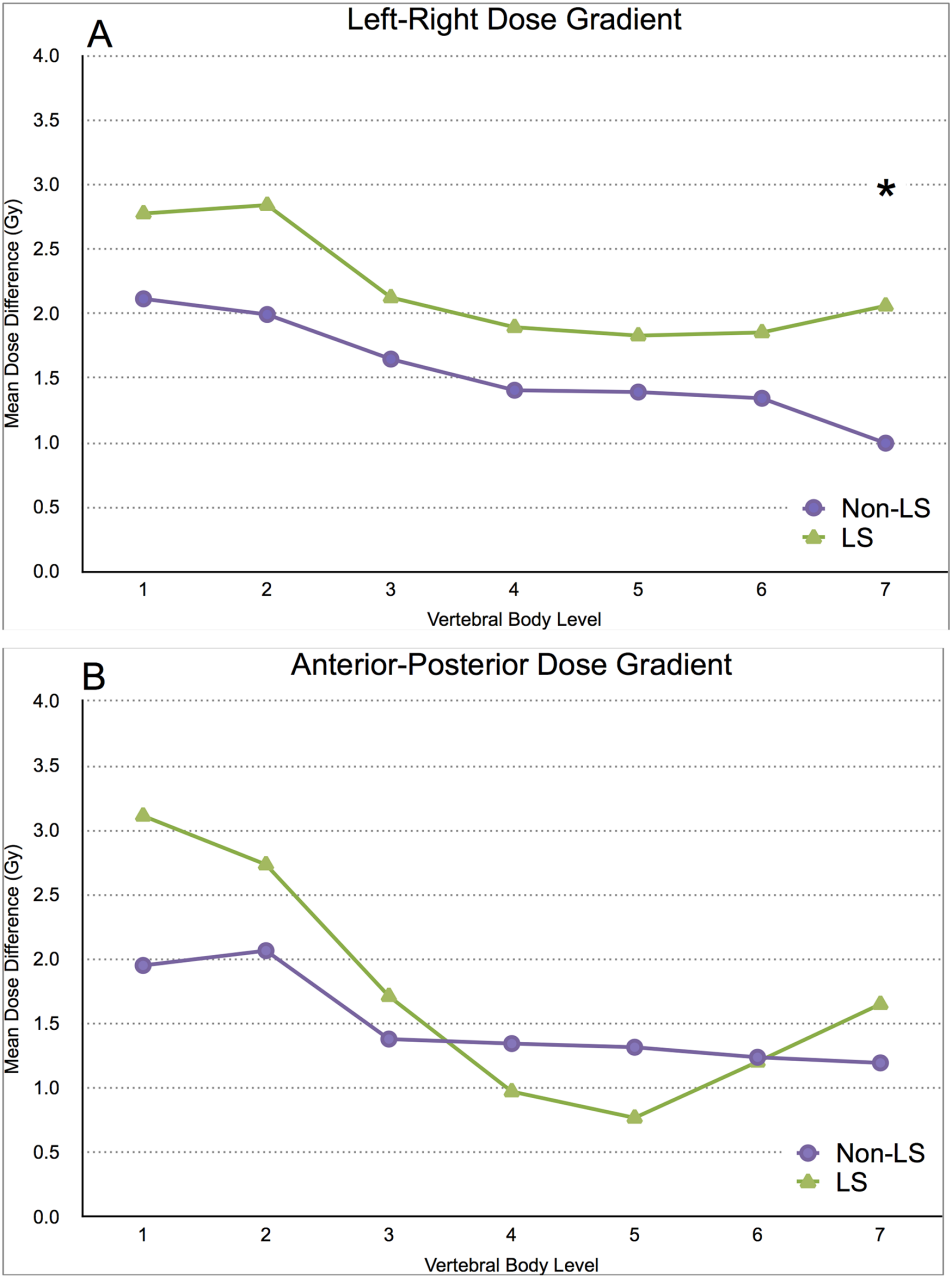
(A) The dose gradient (Gy) between left and right quadrants was calculated in LS (green triangles), and non-LS (purple circles) with respect to cervical spinal level. * A repeated-measures ANOVA (p = 0.026) demonstrate that the LS group means were overall different from those of the non-LS group. (B) The dose gradient (Gy) between anterior and posterior quadrants was calculated in LS (green triangles), and non-LS (purple circles) with respect to cervical spinal level. A repeated-measures ANOVA (p = 0.434) was not significant.

**Fig 2:**
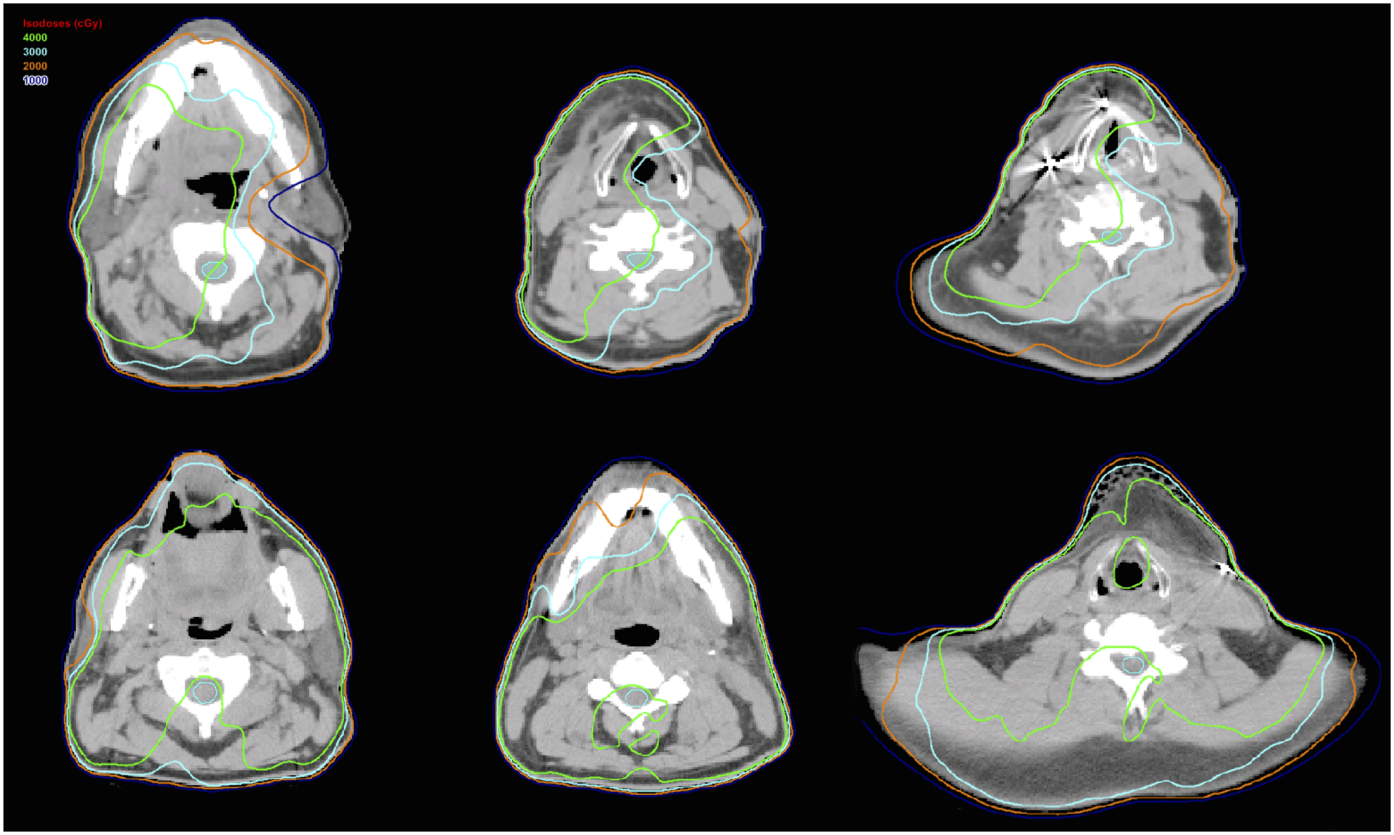
Axial CT images of an LS patient, treated unilaterally (top row), and a non-LS patient, treated bilaterally (bottom row). Note that in the unilaterally treated patient, the 40 Gy line (green) hemisects the spinal cord.

In the lateral direction (left-right), LS patients had a higher dose gradient, over all seven segments of the cervical spine, compared to non-LS counterparts (Fig 1A). Since all LS patients were found to be treated with VMAT unilaterally, the higher lateral dose gradient in these patients is consistent with this observation. This was confirmed by repeated-measures ANOVA (p = 0.030), performed on the left-right mean dose difference, demonstrating that the LS group was overall different from those of the non-LS group. By contrast, the anterior-posterior dose gradient calculation showed that the LS group means were not significantly different from the non-LS group overall, by repeated-measures ANOVA (p = 0.437) Fig 1B). An illustration of the lateral dose gradient with dose distributions on planning CT images of a LS patient and non-LS patient can be found in Fig 2.

**Fig 3:**
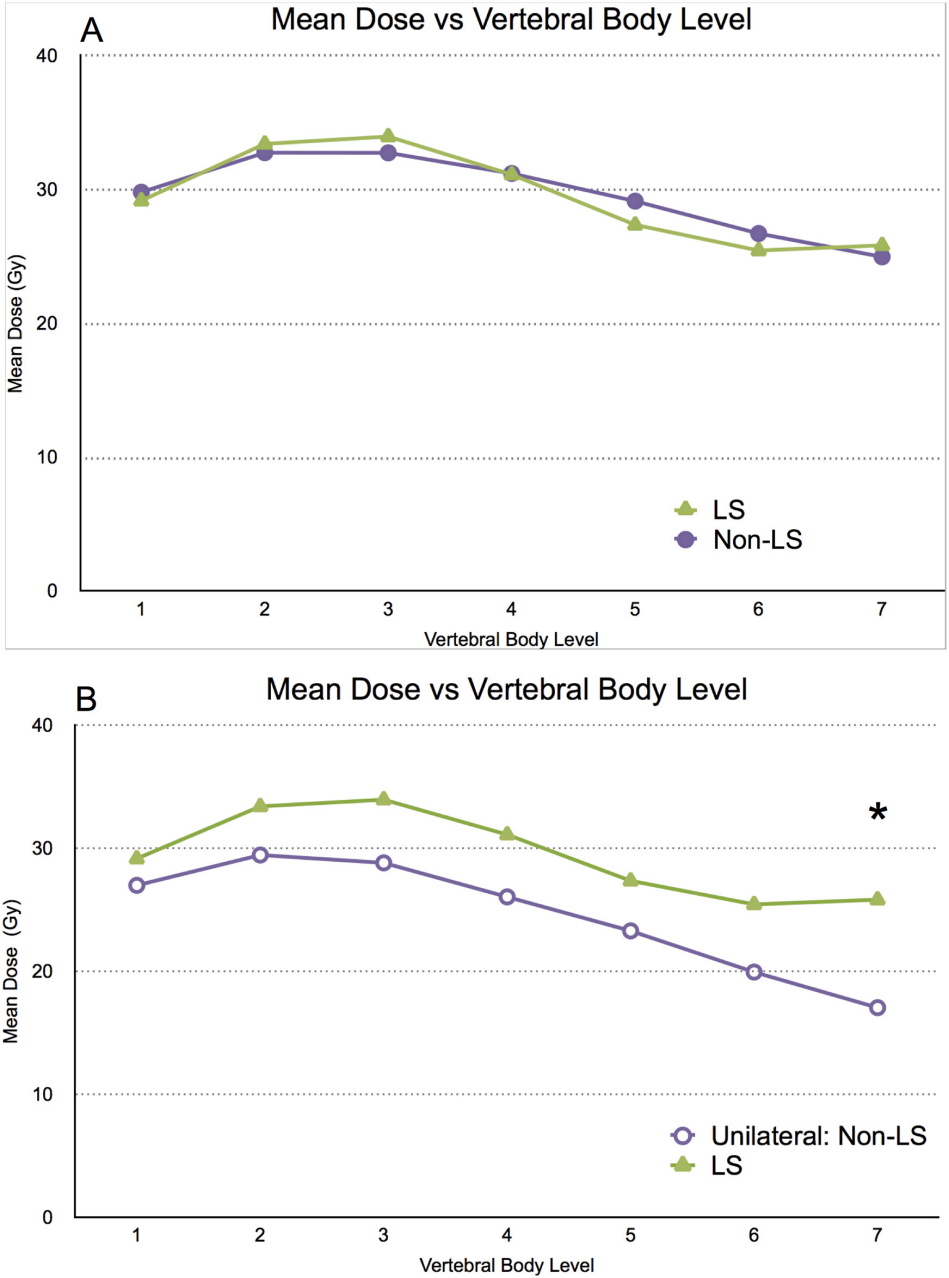
(A) Mean dose (Gy) of LS (green triangles) and non-LS (purple circles) groups with respect to cervical vertebral body level. (B) Mean dose (Gy) of LS (green triangles), and unilateral: non-LS (purple, unfilled circles) groups, with respect to vertebral body level. * A repeated-measures ANOVA (p = 0.035) demonstrated that the LS group means were overall different from those of the unilateral: non-LS group.

The mean dose at each cervical vertebral body level of LS and non-LS patients was similar (Fig 3A). Since all patients that developed LS received radiation unilaterally, we subsequently analyzed the dosimetric parameters within the unilateral treatment group to further elucidate dosimetric characteristics of LS patients (“Unilateral: Non-LS”). In comparing LS patients to unilateral: non-LS patients, we found that LS patients received a higher mean dose across all seven vertebral body levels (Fig 3B), confirmed by repeated-measures ANOVA (p = 0.046), demonstrating that the LS group means were overall different from those of the unilateral: non-LS group. A summary of comparative dosimetric characteristics between LS and non-LS patients within the unilateral treatment group can be found in Table 2.

## Discussion

At our institution, the incidence of LS reported among patients receiving head and neck radiation therapy via VMAT technique is on the higher end of the expected range at 15.2%. Given that this study only looked at retrospective cases, this LS incidence is possibly underestimated due to underreporting, since patients are not routinely questioned regarding LS symptoms. In investigating the clinical characteristics of LS patients at our institution, we found that all LS patients were treated with radiation to one side of the neck. This led to a comparison of the dosimetric parameters between unilateral and bilateral treatment plans, to elucidate any differences that could lead to the development of LS.

While the dosimetric analysis provided a macroscopic view of the radiation-induced injury, we also were led to examine the underlying biological mechanisms through a review of the literature on animal studies. Studies of inhomogeneous radiation dose distribution (bath-and-shower) on the rat cervical spinal cord have been shown to lower the effective dose [16–18], and we believe a similar principle could apply in the human spinal cord. Collectively, this has allowed us to bridge the clinical characteristics of unilateral radiation to the head and neck with the dosimetric patterns of VMAT, guiding us into a mechanistic understanding of radiation-induced LS.

The role of radiation in oligodendrocyte death and white matter regeneration is still not entirely clear. Animal studies have shown that radiation induces glial cell apoptosis in the brain and spinal cord, as early as hours after exposure to radiation [19–23]. These studies have also found that radiation-induced apoptosis is dose-dependent and that radiation causes changes in myelin gene expression [19, 24]. In the weeks after the initial radiation exposure, the white matter begins to recover and the rate of apoptosis declines. By six weeks, the rate of apoptosis and oligodendrocyte cell density in the white matter will approach that of non-irradiated controls, although it has been shown that oligodendrocyte density will not fully recover at higher radiation doses [3, 20]. The existing hypotheses of absolute dose threshold and, later, inhomogeneous dose distribution in the form of a bath-and-shower pattern, have proven to be imperfect as definitive mechanisms of radiogenic LS in patients. It is also thought that a high dose area with a surrounding low dose radiation may inhibit the migration of oligodendrocyte progenitor cells [25]. The migration distance of these re-myelinating cells in the central nervous system has been shown to be 2mm [26], implying that irradiated distances greater than this can lead to impaired re-myelination of denuded areas. Perhaps in conjunction with this effect, radiation may induce the release of an injury signal via inflammatory cytokines [23, 27], the downstream effects of which are unknown.

The only clinical study of bath-and-shower effects by Pak et al. confirmed that higher doses were associated with LS, but did not ultimately find a relationship between low doses seen on the DVH with the incidence of LS. However, the analysis of inhomogeneous dose distribution using the DVH does not take into account the location of the dose in the cervical spinal cord, nor does it account for the degree of inhomogeneity in adjacent regions. Furthermore, all patients in this study were concurrently treated with carboplatin and paclitaxel, each associated with peripheral neuropathy. It perhaps is challenging to differentiate the true effect of a bath-and-shower distribution with chemotherapy as a potential confounder.

**Fig 4:**
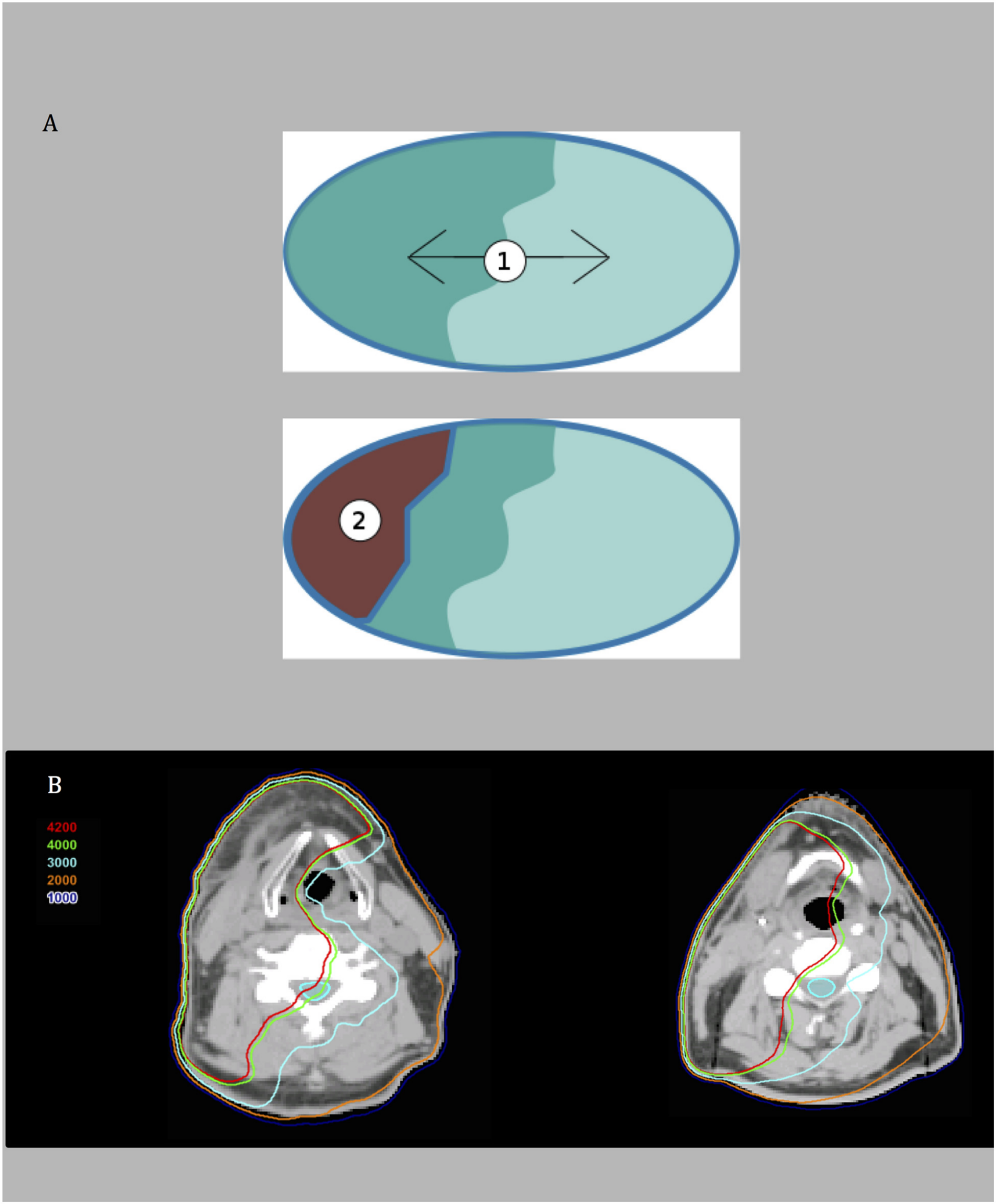
(A) A schematic diagram of an axial slice of cervical spinal cord, illustrating the two-step hypothesis of LS development. 1) A dose gradient is established between adjacent areas of spinal cord in the axial plane. 2) The dark area represents a sufficiently high dose to potentiate the development of LS. (B) Representative axial CT images of dose distribution for an LS patient (left) and non-LS patient (right), both treated unilaterally for cancer of the tonsil. In the LS patient (left), the cervical spine is exposed to both a dose gradient and near tolerance doses (40 Gy represented by green line). By contrast, in the non-LS patient (right), there is no dose gradient with the cord completely bathed in 30 Gy (light blue line).

We postulate that the inhomogeneous dose distribution can be evaluated through axial slices of the treated spinal cord, rather than through the DVH alone. The dose gradient can represent the level of dose inhomogeneity in the axial plane. We propose that the high level of conformality achieved by VMAT confers a steep dose gradient in the axial plane. Higher dose gradients between two adjacent portions of a given axial slice of cervical spine would create an inhomogeneous, bath-and-shower dose distribution as a setting for impaired oligodendrocyte progenitor migration. Dose distribution in the axial plane may be particularly relevant if progenitor cell migration occurs from the spinal region contralateral to oligodendrocyte cell death. However, a dose gradient alone may not be sufficient to cause LS; dose threshold could be the second factor contributing to the development in LS. This is supported by the appearance of higher mean doses to the cervical spinal cord in the LS group compared to non-LS patients within the unilateral treatment group. That is, 1) an inhomogeneous dose distribution (bath-and-shower) with 2) a sufficiently high dose, may be the specific environment which potentiates the development of radiogenic LS in head and neck cancer patients. The dose gradient may serve to sensitize the tissue to high doses of radiation Fig 4A illustrates this two-step hypothesis. For the LS patient (Fig 4B, left): the upper cervical spine is exposed to both 1) a dose gradient along the axial plane with right and left cord hemisected by 40 Gy isodose line and 2) the right side of the cord at these levels is exposed to near tolerance doses (42 Gy). By contrast (Fig 4B, right), a similar case of a unilaterally treated non-LS patient, showing that the cord is completely bathed in 30 Gy with no dose gradient.

A few major limitations in this study were the retrospective nature and small series, making it difficult to draw strong conclusions. However, given that so little is known about the process of radiation-induced LS, we believe that the high incidence of LS in this specific treatment group will serve an important role in raising awareness within the radiation oncology community to screen and counsel patients for LS. Further, the potential roles of spinal cord dose gradient and inhomogeneous dose distribution can have implications that extend beyond LS, providing insights into the mechanisms involved in spinal cord toxicity and ripe ideas for future research. Practicing radiation oncologists should consider counseling head and neck patients for LS, particularly after unilateral VMAT-based radiation, as this is a frightening side effect that is largely temporary and recoverable.

Further examination of dosimetric factors, dose difference, magnitude, or contiguous length of cord irradiated, or some combination thereof, is warranted to determine treatment-planning parameters associated with development of LS in this setting. Going forward, we hope that continued efforts in this area of research can help elucidate the pathophysiology underlying the development of LS, and allow us to more effectively screen and counsel patients for LS as a side effect of radiation therapy.
